# Hypertension-Mediated Organ Damage Correlates With Serum Homocysteine Level in Community-Dwelling Elderly Chinese: The North Shanghai Study

**DOI:** 10.3389/fcvm.2021.662741

**Published:** 2021-05-10

**Authors:** Zhongyuan Ren, Jun Zhang, Shikai Yu, Song Zhao, Jiamin Tang, Yixing Zheng, Weilun Meng, Chong Xu, Yi Zhang, Yawei Xu

**Affiliations:** ^1^Department of Cardiology, Shanghai Tenth People's Hospital, Tongji University School of Medicine, Shanghai, China; ^2^Soochow University Medical College, Suzhou Industrial Park (SIP), Suzhou, China

**Keywords:** homocysteine, hypertension-mediated organ damage, hyperhomocysteinemia, Chinese, elderly

## Abstract

**Introduction:** Serum homocysteine (Hcy) level is associated with cardiocerebrovascular disease. However, the relationship between Hcy and hypertension-mediated organ damage (HMOD) in non-hospitalized residents has not been elucidated. We aimed to investigate the association of HMOD with Hcy in elderly Chinese.

**Methods:** One thousand seven hundred and forty-four community-dwelling elderly Chinese (age ≥65 years) participated in the Northern Shanghai Study from Jun. 2014 to Aug. 2015. Hyperhomocysteinemia (HHcy) was defined as serum Hcy ≥15 mmol/L, and HMOD was estimated as arterial stiffness [carotid-femoral pulse wave velocity (CF-PWV) and ankle-brachial index (ABI)], cardiac impairment [left ventricular (LV) hypertrophy and LV diastolic dysfunction], and renal dysfunction [estimated glomerular filtration rate (eGFR) and urinary albumin/creatinine ratio]. Linear and logistic regression models were built to explore the associations of HMOD with Hcy.

**Results:** Among 1,744 participants, 632 (36.2%) were diagnosed as HHcy. HHcy group had more men (61.2 vs. 35.3%), with higher age (73.7 ± 6.7 vs. 70.4 ± 5.3 years) and BMI (24.2 ± 3.4 vs. 23.7 ± 3.5 kg/m^2^). Linear regression analysis showed that serum Hcy level was positively associated CF-PWV and negatively associated with ABI and eGFR. By logistic regression, HHcy was significantly associated with abnormal CF-PWV [odds ratio (OR) = 1.53, 95% confidence interval (CI) 1.08–2.16] and ABI (OR = 1.55, 95% CI 1.17–2.04), and decreased eGFR (OR = 7.09, 95% CI 4.03–12.47) after adjustment for covariates. Moreover, similar associations of serum Hcy level with CF-PWV and eGFR were observed in subgroups by gender and hypertensive state.

**Conclusion:** HMOD, particularly renal dysfunction and arterial stiffening, was significantly and independently associated with increased serum Hcy level in the elderly Chinese.

**Clinical Trial Registration:** [ClinicalTrial.gov], identifier [NCT02368938].

## Introduction

Homocysteine (Hcy) is an intermediate product of cellular methylation process and can be converted to methionine (Met) *via* a reaction facilitated by vitamin B12 and folate. Genetic defect or folate deficiency could lead to pathological accumulation of Hcy in the serum that defined as hyperhomocysteinemia (HHcy). HHcy has been proven to be associated with a large variety of diseases, including neural tube defect (1), cognitive impairment (2), and cardiovascular disease (CVD) (3).

The association of HHcy and hypertension has long been a topical issue. Previous studies have validated that serum Hcy level correlates with blood pressure (BP) (4–6). However, the relationship could be greatly influenced by multiple factors such as race, gender, age, and nutritional supplementation (7). Recently, a large longitudinal study done by Tao et al. (8) proved that serum Hcy level predicts hypertension. With a high prevalence of hypertension (9) and HHcy (7), Hcy might play a more important role in Chinese population.

Besides, HHcy has been recognized as an independent risk factor of CVD. Early in 1969, Dr. Kilmer S. McCully (10) had already demonstrated the vascular lesion in a child with HHcy. Hitherto, HHcy has been proven to be associated with various hypertension-mediated target organ damage (HMOD), including atherosclerosis (11), cardiac arrhythmia (12), stroke (13), and renal dysfunction (14). However, the comprehensive evaluation of HHcy and HMOD has only been validated in hospitalized patients with chronic kidney disease (CKD) (15) and diabetes mellitus (DM) (16). Because both HHcy and HMOD can be subclinical, a thorough investigation of Hcy and HMOD in general population is warranted.

In this study, we aimed to comprehensively investigate the association of serum level of Hcy and HHcy with HMOD, based on a cohort of 1,744 community-dwelling elderly Chinese from the Northern Shanghai Study.

## Materials and Methods

### Study Population

A total of 1,744 subjects with Hcy data available derived from the Northern Shanghai Study were included for the present study. The Northern Shanghai Study is an ongoing prospective and registered study (ClinicalTrial.gov Identifier NCT02368938) and has been described in detail in our previous articles (17, 18).

The inclusion criteria of the Northern Shanghai Study were: (1) age ≥65 years; (2) local residents from urban communities in the north of Shanghai, and (3) available for long-term follow-up. Meanwhile, the exclusion criteria were: (1) severe cardiac insufficiency [New York Heart Association (NYHA) class IV] or severe renal insufficiency (stage 5 chronic kidney disease); (2) history of stroke within 3 months; and (3) malignant tumor. Inform of consent was obtained from every participant. The study was approved by the ethics committee of Shanghai Tenth People's Hospital.

### Medical History, Anthropometrics, and Laboratory Tests

An organized questionnaire was used to collect the medical history and familial history of each participant, encompassing present illness, smoking status, and history pre-existing diseases such as hypertension, DM, and cardiovascular, cerebrovascular and renal diseases. All the information was collected by well-trained physicians at community healthcare center before any measurement.

With height and weight measured, body mass index (BMI) was calculated as weight (kg) divided by squared height (m^2^). BP was measured by well-trained physicians in a room with standard temperature (22–27°C). Participants were required to sit upright when brachial BP was measured by a mercury sphygmomanometer. BP for each participant was measured for three times, with an interval of 5 min. Hypertension was defined as measured average systolic BP ≥ 140 mmHg or diastolic BP ≥ 90 mmHg or taking any antihypertensive agents.

Blood and urine samples of each participant were analyzed in the Department of Laboratory Medicine of Shanghai Tenth Peoples' Hospital by experienced technicians. Blood and urine routine tests were conducted, and biochemical measurement of serum Hcy, blood glucose, glycated hemoglobin, lipid metabolism markers, blood and urinary creatinine, and urinary albumin excretion were examined. Diagnosis of DM was confirmed when fasting serum glucose ≥7.0 mmol/L or referred to self-reported antidiabetic medication. HHcy was defined as serum level of Hcy ≥15.0 μmol/L (19).

### Evaluation of HMOD

#### Cardiac Impairment

Transthoracic echocardiography (TTE) was performed by two experienced physicians using MyLab 30 CB machine (ESAOTE SPA). In the parasternal long-axis view in M-mode, left ventricular end-diastolic diameter (LVEDd), interventricular septal diameter (IVSd), posterior wall thickness end-diastole diameter (PWTd), and left ventricular end-systolic diameter (LVESd) were indices measured, which is in accordance with the guidelines of American Society of Echocardiography (ASE) (20). Left ventricular mass index (LVMI) was calculated by the formula: LVMI (g/m^2^) = (0.8 × 1.04 × [(LVEDd+PWTd+IVSd)^3^-(LVEDd)^3^] +0.6) / BSA (body surface area, m^2^). Left ventricular hypertrophy was defined as LVMI ≥ 115 g/m^2^ for male, or ≥95 g/m^2^ for female. In the apical four-chamber view, peak early diastolic transmitral flow velocity (E) and early diastolic lateral mitral annular velocity (Ea) were measured with pulse wave and tissue Doppler imaging, respectively, and the ratio of E/Ea was then calculated. Left ventricular diastolic dysfunction (LVDD) was considered present when E/Ea ≥ 15 or 15>E/Ea>8 mean while LVMI>149 g/m^2^ for male or LVMI ≥ 122 g/m^2^ for female (21, 22).

#### Arterial Stiffness

**Ankle-brachial index (ABI)** was defined as the brachial systolic BP divided by ankle systolic BP that measured by the VP1000 system (Omron, Tokyo, Japan). Bilateral ABI was measured and calculated concurrently. ABI ≤ 0.9 was considered as significant arterial damage.

Bilateral **carotid plague and carotid intima-media thickness (IMT)** were evaluated by ultrasonography using MyLab 30 Gold CV system (ESAOTE SpA, Genoa, Italy). Carotid plaque was defined as increment of IMT > 50% of the surrounding wall thickness or IMT>1.5 mm on at least one side, and the abnormal IMT was defined as IMT>0.9 mm (23).

**Carotid-femoral pulse wave velocity (CF-PWV)** was defined as pulse wave travel distance divided by the travel time. By two well-trained physicians, ipsilateral carotid and femoral pulse waves were recorded using applanation tonometry (SphygmoCor; AtCor Medical, Sydney, Australia). The superficial distances from the sternal notch to the right carotid artery and from the sternal notch to the right femoral artery were measured, and then the traveling distance of pulse wave was calculated as the difference between the two measured distances. With pulse wave travel time automatically recorded, CF-PWV was automatically calculated by the device. Quality of measurement was guaranteed with an operator index > 80%. Arterial stiffness was indicated when CF-PWV > 10 m/s.

#### Renal Dysfunction

Renal dysfunction was assessed by urinary albumin-creatinine ratio (UACR) and estimated glomerular filtration rate (eGFR). eGFR was calculated according to the modified MDRD (modification of diet in renal disease) formula: eGFR (mL/min/1.73 m^2^) =175 × PCr^−1.234^ × age^−0.179^ (women × 0.79). While UACR was calculated as urinary microalbumin level divided by urinary creatinine level. UACR ≥ 30 mg/g and eGFR ≤ 60 mL/min/1.73 m^2^ was stipulated as renal damage.

### Statistical Analysis

Values were presented as mean ± standard deviation or numbers and percentages in parenthesis for quantitative and qualitative variables, respectively. The student *t*-test and Chi-square test were applied to detect the differences between two groups with and without HHcy. The correlation of cardiovascular risk factors with Hcy, and HMOD with Hcy were investigated by Spearman if the parameter was continuous or Kendall's correlation analysis if parameter categorized. Crude and adjusted linear regression analysis were conducted to explore the association of HMOD with serum Hcy level, while logistic regression analysis was adopted to estimate the association of HMOD with HHcy. ANOVA was applied to compare the difference of CF-PWV and eGFR among four quartiles of Hcy. A two-tailed *P*-value < 0.05 was considered significant. Statistical analysis system (SAS) version 9.4 (SAS Institute Inc.) was used for all the statistical analysis.

## Results

### Baseline Characteristics

Among 1,744 participants, there were 632 (36.2%) participants with HHcy. Compared with participants without HHcy, those with HHcy was composed of more male (61.2 vs. 35.3%, *P* < 0.001) and smokers (34.7 vs. 17.6%, *P* < 0.001), and had significantly higher age (73.7 ± 6.7 vs. 70.4 ± 5.3 years, *P* < 0.001) and BMI (24.2 ± 3.4 vs. 23.7 ± 3.5 kg/m^2^, *P* = 0.01), but less DM (16.1 vs. 21.2%, *P* = 0.001), and lower high-density lipid cholesterol (HDL-C) level (1.3 ± 0.4 mmol/L vs. 1.4 ± 0.4 mmol/L, *P* < 0.001). While there are more diabetic participants in the normal Hcy group (21.2%). In terms of hypertension-mediated organ damage, HHcy group had generally severer arterial impairment including thicker carotid intima-medium (IMT, 0.63 ± 0.16 mm vs. 0.60 ± 0.14 mm, *P* = 0.002), higher carotid-femoral pulse wave velocity (PWV, 9.8 ± 2.5 m/s vs. 9.1 ± 2.1 m/s, *P* < 0.001), and higher proportion of low ankle brachial index (ABI, 25.4 vs. 13.8%, *P* < 0.001). And worse renal function including lower estimated glomerular filtration rate (eGFR, 82.2 ± 20.8 mL/min/1.73 m^2^ vs. 99.2 ± 20.3 mL/min/1.73 m^2^, *P* < 0.001) and higher UACR (65.2 ± 122.5 mg/g vs. 50.6 ± 87.4 mg/g, *P* = 0.001). Echocardiographic data was not available for 9 participants due to refusal of echocardiography examination. Owning to the dominance of male in HHcy group, higher Left ventricular mass index (LVMI, 93.3 ± 30.9 g/m^2^ vs. 88.8 ± 27.8 g/m^2^, *P* = 0.002) but similar proportion of LVH and LVDD were observed. Detailed information was listed in Table 1.

**Table 1:** Characteristics of study population grouped by hyperhomocysteinemia (HHcy).

**Variables**	**Total (*N* = 1,744)**	**HHcy**		***P***
		**No (*N* = 1,112, 63.8%)**	**Yes (*N* = 632, 36.2%)**	
**Cardiovascular risk factors**				
Age, years	71.4 ± 6.1	70.4 ± 5.3	73.7 ± 6.7	**<0.001**
Male gender, *n* (%)	779 (44.7)	392 (35.3)	387 (61.2)	**<0.001**
BMI, kg/m^2^	23.9 ± 3.5	23.7 ± 3.5	24.2 ± 3.4	**0.010**
Smoking, *n* (%)	415 (23.8)	196 (17.6)	219 (34.7)	**<0.001**
Diabetes, *n* (%)	337 (19.3)	235 (21.2)	102 (16.1)	**0.001**
HDL-C, mmol/L	1.4 ± 0.4	1.4 ± 0.4	1.3 ± 0.4	**<0.001**
LDL-C, mmol/L	3.2 ± 0.9	3.2 ± 0.9	3.2 ± 0.9	0.592
FH of Premature CVD, *n* (%)	373 (21.5)	249 (22.5)	124 (19.7)	0.181
**HMOD parameters**				
LVMI, g/m^2^	90.4 ± 29.0	88.8 ± 27.8	93.3 ± 30.9	**0.002**
LVH, *n* (%)	481 (27.6)	317 (28.5)	164 (26.0)	0.251
E/Ea	9.7 ± 3.6	9.7 ± 3.5	9.7 ± 3.8	0.689
LVDD, *n* (%)	233 (13.4)	148 (13.3)	85 (13.5)	0.806
Carotid plaque, *n* (%)	628 (36.1)	725 (65.3)	445 (70.9)	**0.018**
IMT, mm	0.61 ± 0.15	0.60 ± 0.14	0.63 ± 0.16	**0.002**
IMT > 0.9 mm, *n* (%)	71 (4.1)	37 (3.3)	34 (5.4)	**0.036**
CF-PWV, m/s	9.4 ± 2.3	9.1 ± 2.1	9.8 ± 2.5	**<0.001**
CF-PWV > 12 m/s, *n* (%)	196 (11.6)	98 (9.0)	98 (16.4)	**<0.001**
ABI ≤ 0.9, *n* (%)	306 (17.9)	151 (13.8)	155 (25.4)	**<0.001**
eGFR, mL/min/1.73m^2^	92.9 ± 22.0	99.2 ± 20.3	82.2 ± 20.8	**<0.001**
eGFR <60, *n* (%)	102 (5.9)	17 (1.6)	85 (13.1)	**<0.001**
UACR, mg/g	55.7 ± 100.8	50.6 ± 87.4	65.2 ± 122.5	**0.001**
UACR > 30, *n* (%)	736 (43.5)	455 (42.2)	281 (45.7)	0.164

*Values are presented as mean ± standard deviation or numbers and percentages in parenthesis for quantitative and qualitative variables, respectively. The student t-test and Chi-square test were applied to detect the differences between two groups*.

*HHcy, hyperhomocysteinemia; BMI, body mass index; HDL-C, high-density lipid cholesterol; LDL-C, low-density lipid cholesterol; FH of premature CVD, familial history of premature cardiovascular diseases; HMOD hypertension-mediated organ damage; LVMI, left ventricular mass index; LVH, left ventricular hypertrophy; E/Ea, ratio of peak early diastolic transmitral flow velocity (E) and early diastolic lateral mitral annular velocity (Ea); LVDD, left ventricular diastolic dysfunction; IMT, carotid intima-media thickness; ABI, ankle-brachial index; CF-PWV, carotid-femoral pulse wave velocity; eGFR, estimated glomerular filtration rate; UACR, urinary microalbumin/creatinine ratio. Numerical data in bold indicates significant value with P < 0.05*.

As we performed Subgroup analysis in patients with HHcy and E/e' below and above 15, elder age (75.4 ± 6.9 years vs. 72.8 ± 6.7 years, *P* = 0.008), higher BMI (25.2 ± 3.3 kg/m^2^ vs. 24.1 ± 3.4 kg/m^2^, *P* = 0.019), and higher LVMI (103.7 ± 34.3 g/m^2^ vs. 92.0 ± 30.2 g/m^2^, *P* = 0.008) were observed in the group of E/e'>15.

### The Association of HMOD With Hcy and HHcy

Correlation analysis showed independent correlation between HHcy and age [Correlation coefficient (*r*) = 0.19, *P* < 0.001], gender [*r* = 0.25, *P* < 0.001], BMI [*r* = 0.08, *P* = 0.001], smoking status [*r* = 0.19, *P* < 0.001], DM [*r* = −0.06, *P* < 0.011], and HDL level [*r* = −0.17, *P* < 0.001]. For HMOD indices, LVMI [*r* = 0.06, *P* = 0.002], IMT [*r* = 0.07, *P* = 0.002], CF-PWV [*r* = 0.14, *P* < 0.001], ABI [*r* = 0.15, *P* < 0.001], and eGFR [*r* = −0.38, *P* < 0.001] were significantly correlated with HHcy. Results of HHcy and other variables were showed in Table 2.

**Table 2:** The correlation between HHcy and cardiovascular risk factors and HMOD parameters.

**Variables**	**Correlation coefficients (*r*)**	***P*-values**
**Cardiovascular risk factors**		
Age, years	0.190	**<0.001**
Gender (female = 0, male = 1)	0.250	**<0.001**
Body mass index, kg/m^2^	0.079	**0.001**
Smoking (no = 0, yes = 1)	0.190	**<0.001**
Diabetes (no = 0, yes = 1)	−0.061	**0.011**
HDL, mmol/L	−0.170	**<0.001**
LDL, mmol/L	−0.024	0.322
Familial history of Premature CVD	−0.032	0.182
**HMOD parameters**		
LVMI, g/m^2^	0.060	**0.002**
E/Ea	−0.024	0.331
IMT, mm	0.070	**0.002**
CF-PWV, m/s	0.140	**<0.001**
ABI (≤ 0.9 = 0, >0.9 = 1)	0.150	**<0.001**
eGFR, mL/min/1.73 m^2^	−0.380	**<0.001**
UACR, mg/g	0.038	0.120

*Spearman or Kendall's correlation analysis was used to investigate the correlation between HHcy and cardiovascular risk factors and organ damage variables. Correlation coefficients and the corresponding p-values were shown in the table*.

*HMOD, hypertension-mediated organ damage. Numerical data in bold indicates significant value with P < 0.05*.

The association of serum Hcy level with HMOD was evaluated by linear regression analysis. Unadjusted linear regression showed HMOD parameters including LVMI, E/Ea, IMT, CF-PWV, ABI, eGFR, and UACR were significantly associated with serum Hcy level. After adjusted by cardiovascular risk factors, only CF-PWV (β = 0.08), ABI (β = −0.11), eGFR (β = −0.36) remained significant. Results were listed in Table 3.

**Table 3:** Independent association of HMOD with serum Hcy level.

**HMOD parameters**	**Model^a^**		**Model^b^**		**Model^c^**	
	**β**	***P***	**β**	***P***	**β**	***P***
LVMI	0.001	**0.001**	<0.001	0.052	<0.001	0.109
E/Ea	−0.001	0.141	< −0.001	0.325	−0.001	0.217
IMT	0.055	**0.019**	−0.010	0.653	−0.017	0.449
CF-PWV	0.150	**<0.001**	0.090	**0.025**	0.080	**0.0016**
ABI	−0.180	**<0.001**	−0.130	**<0.001**	−0.110	**<0.001**
eGFR	−0.410	**<0.001**	−0.380	**<0.001**	−0.360	**<0.001**
UACR	0.030	0.292	0.010	0.305	0.003	0.918

*Linear regression analysis was conducted to investigate the association of hcy and HMOD. Logarithmic transformation was applied to serum Hcy level in order to comply normal distribution. Regression coefficients (β) and the corresponding p-values derived from different models were presented in the table*.

a *Model a was not adjusted*.

b *Model b was adjusted for age, gender, and body mass index*.

c *Model c was adjusted for smoking, diabetes, hypertension, high-density lipid cholesterol, low-density lipid cholesterol, familial history of premature cardiovascular diseases, and the variables in Model b. Numerical data in bold indicates significant value with P < 0.05*.

Logistic regression analysis was further conducted to explore the association between HMOD and HHcy, as shown in Table 4. After adjusted for all potential covariates, increased CF-PWV (OR = 1.53, 95%CI 1.08, 2.16), decreased ABI (OR = 1.55, 95%CI 1.17, 2.04), and eGFR (OR = 7.09, 95%CI 4.03, 12.47) were significantly associated with HHcy, respectively.

**Table 4:** Independent association of HMOD with HHcy.

**HMOD parameters**	**OR (95%CI)^a^**	**OR (95%CI)^b^**	**OR (95%CI)^c^**
LVH, (no = 0, yes = 1)	0.88 (0.71, 1.10)	1.02 (0.79, 1.30)	1.00 (0.78, 1.29)
LVDD, (no = 0, yes = 1)	1.01 (0.76, 1.35)	1.10 (0.80, 1.50)	1.04 (0.76, 1.44)
IMT > 0.9 mm, (no = 0, yes = 1)	1.60 (0.36, 2.82)	1.36 (0.82, 2.28)	1.26 (0.74, 2.15)
CF-PWV > 12 m/s, (no = 0, yes = 1)	**1.98 (1.70, 2.67)**	**1.47 (1.05, 2.05)**	**1.53 (1.08, 2.16)**
ABI ≤ 0.9, (no = 0, yes = 1)	**2.12 (1.66, 2.74)**	**1.60 (1.22, 2.09)**	**1.55 (1.17, 2.04)**
eGFR <60 mL/min/1.73 m^2^, (no = 0, yes = 1)	**9.95 (5.85, 16.92)**	**7.07 (4.06, 12.32)**	**7.09 (4.03, 12.47)**
UACR > 30, (no = 0, yes = 1)	1.15 (0.94, 1.41)	1.11 (0.89, 1.37)	1.10 (0.88, 1.37)

*Logistic regression analysis was conducted to investigate the association of HHcy and HMOD. Odds Ratio with 95% confidence interval [OR (95%CI)] derived from different models were presented in the table*.

a *Model a was not adjusted*.

b *Model b was adjusted for age, gender and body mass index*.

c *Model c was adjusted for smoking, diabetes, hypertension, high-density lipid cholesterol, low-density lipid cholesterol, familial history of premature cardiovascular diseases, and the variables in Model b*.

*LVH, left ventricular hypertrophy; LVDD, left ventricular diastolic dysfunction; IMT, carotid intima-media thickness; ABI, ankle-brachial index; CF-PWV, carotid-femoral pulse wave velocity; eGFR, estimated glomerular filtration rate; UACR, urinary microalbumin/creatinine ratio. Numerical data in bold indicates significant value with P < 0.05*.

### Subgroup Analysis

**Figure 1:**
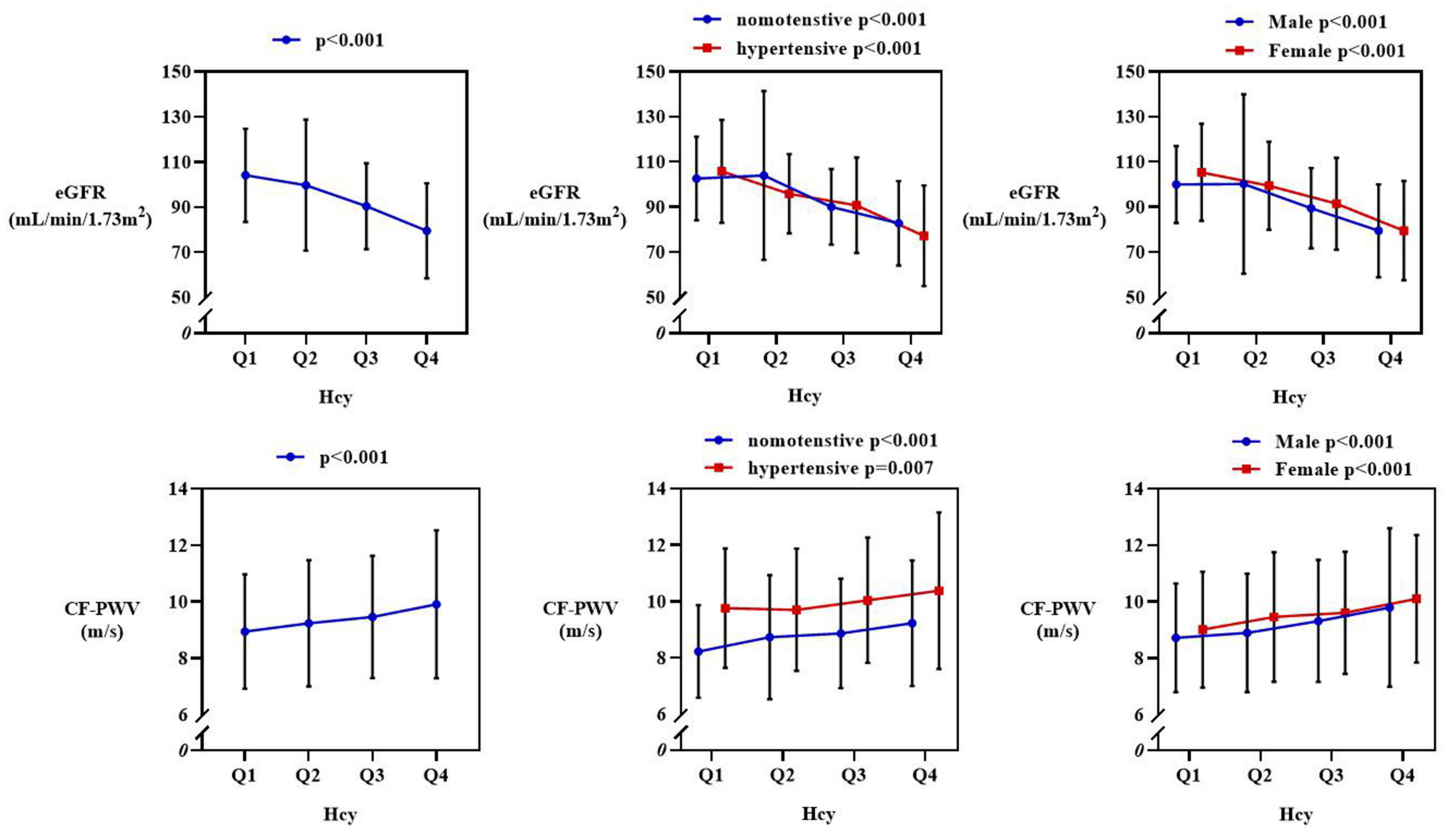
Subgroup analysis of estimated glomerular filtration rate (eGFR) and carotid-femoral pulse wave velocity (CF-PWV) from Q1 to Q4 quartile of Hcy. The row above showed a significant drop of eGFR, and it remained significant in subgroups divided by hypertensive state and gender. The row below showed an increasing pattern of CF-PWV, and it stayed significant in subgroups.

As we further evaluated CF-PWV and eGFR levels among four quartiles of Hcy, CF-PWV increased significantly (*p* < 0.001) and eGFR dropped significantly (*p* < 0.001). Such tendencies remained significant in subgroups divided by gender or hypertensive state. Subgroup analysis was depicted in Figure 1.

## Discussion

Our study comprehensively investigated the association of HMOD with serum Hcy level and HHcy in 1,744 community dwelling Chinese, our major findings are: 1. High Hcy level and HHcy associated with severer HMOD, especially arterial stiffness (indicated by CF-PWV) and renal dysfunction (indicated by eGFR), 2. Such association remained significant in subgroups divided by gender and hypertensive state.

The relationship between Hcy and hypertension has been disputable. As far as we know, serum Hcy level is influenced by complex factors such as gender, age, race, and nutrition supplementation, and these factors confound the results to some extent. Prospective studies on Caucasians have validated that there is no causal relationship between Hcy and hypertension (24, 25). In 2017, a meta-analysis incorporating 15 studies worldwide also showed the positive causal relationship might be confounded by several factors (26). However, in 2018 Tao et al. (8) described the gender-specific causal relationship based on a sample of 3,913 participants from a longitudinal cohort in Chinese population. Other studies from China also demonstrated positive relationship between Hcy level and hypertension (27–29). According to current evidence, we postulate that ethnic disparity accounts for the opposite conclusions between Asian and people of other ethnics and that Hcy could possibly play a more important role in CVD in Chinese population. Therefore, exploration of the association between Hcy and underlying organ damage could be of great importance to better understand the interplay of each other.

Elevated serum Hcy level is both an indicator of underlying inflammatory process and a proinflammatory factor *per se*. Hcy is associated with inflammatory cytokines and in patients with CVD. In a study of 4,164 patients with suspected stable angina pectoris by Bjørnestad et al., serum level of Hcy was a predictive factor for acute myocardial infarction only when accompanied by elevated neopterin, indicating inflammation could be the potential pathogenic process that aggravates CVD and produces Hcy (30). Besides, elevated Hcy level also reveals reduced antioxidative capacity. Methionine–homocysteine cycle is affected by redox status which plays an crucial role in vascular impairment (31). Moreover, intense oxidative stress could inhibit the activity of 5-methyltetrahydrofolate and vitamin B12, which eventually results in accumulated Hcy (32). More importantly, pathological accumulated Hcy *per se* precipitates CVD as they lead to various HMOD. Serum Hcy could directly damage endothelium through interfering the nitric oxygen (NO) production and prompting oxidative stress, and eventually progress into atherosclerosis (33). Collectively, prior investigations show that inflammation and oxidative capacity are strongly related to Hcy, while the specific interaction yet remained to be elucidated.

From clinical aspect, many studies have validated that HHcy is associated with diseased result from atherosclerosis, including ischemic heart disease (10, 34, 35), stroke (13), and atherosclerotic renal dysfunction (14). Moreover, the serum Hcy level is also associated with preclinical indexes of arterial stiffness like PWV (36, 37). Similarly, in our study, we found that a higher level of Hcy and the presence of HHcy is associated with PWV, and a significant positive correlation can be observed in subgroups divided by gender or hypertensive state. We conclude that serum Hcy level and HHcy could be a valuable indicator for arterial stiffness independent of gender and hypertensive state. And this could help stratify residents base on the risk of vascular events, thus improve the prevention of cardiovascular disease.

Besides, serum Hcy level is affected by the severity of renal dysfunction, vice versa. Hcy is primarily excreted through liver and kidney. Thus, deteriorated renal function will cause abnormal accumulation of Hcy. Meanwhile accumulated Hcy will lead to atherosclerosis and in turn impairs renal function, and subsequently trapped into a viscous circle. Studies have shown that the prevalence of HHcy in CKD patients is higher than that of normal population (37). Furthermore, Levi et al. (14) observed the impact of HHcy on renal function through 5-year follow-up of 3,602 participants, and they found HHcy is an independent risk factor of decline of renal function. In our study, we observed similar association of serum Hcy level and HHcy with renal function. Collectively, current evidences support that Hcy is not only associated with renal function, but the accumulation of it poses a higher risk of developing renal dysfunction.

Last but not the least, the association between serum Hcy level and HHcy and left ventricular hypertrophy necessitates further discussion. HHcy has been validated to be associated with left ventricular (LV) structural changes in hypertensive patients (38) and DM patients (16, 39). Besides, patients with concomitant HHcy and metabolic syndrome has a stronger association between HHcy and LVMI than those without metabolic syndrome (40). However, there is a lack of evidence in general population. We found no association between Hcy level and HHcy and cardiac damage (indicated by LVMI and LVDD) in our community dwelling elderly population, whether adjusted or not. As we know, DM and hypertension could lead to LV hypertrophy independent of Hcy level (41). While currently no direct mechanism of cardiac lesion caused by Hcy has been proposed. Hence, we believe the high occurrence of cardiac damage in HHcy patients is mostly contributed either by vascular lesion or concomitant diseases rather than Hcy *per se*. Whether Hcy could cause and aggravate cardiac damage pers se necessitates further longitudinal researches.

This is an observational, cross-sectional study that cannot validate the causal relationship between serum Hcy level and HHcy and HMOD. Further longitudinal studies are warranted to comprehensively prove the relationship. Besides, the supplementation and of vitamin B6 and B12 and folate of each individual was not collected. Nutritional supplementation has been proven that it not only lower Hcy level but decrease the incidence of CVD. However, no folate fortification had been carried out in any of the communities. Future studies Focusing on the relationship amnog the nutrition supply, Hcy and CVD are warranted. Last but not least, the definition of LVDD in the Northern Shanghai Study was defined according to the old definition (21, 22) which is different from the most updated definition of LVDD from ESC guideline (42). Therefore, our results related to LVDD should be interpreted with care. Future studies based on the new LVDD definition are needed to confirm our findings.

## Data Availability Statement

The original contributions presented in the study are included in the article, further inquiries can be directed to the corresponding author/s.

## Ethics Statement

The studies involving human participants were reviewed and approved by ethics committee of Shanghai Tenth People's Hospital. The patients/participants provided their written informed consent to participate in this study. Written informed consent was obtained from the individual(s) for the publication of any potentially identifiable images or data included in this article.

## Author Contributions

ZR and SY contributed to the interpretation of data for the work. ZR, JZ, and SY contributed to drafting the work. SZ, JT, YZhe, WM, and CX contributed to the acquisition of data. YZha and SY contributed to the analysis and revision of the work. YZha and YX contributed to the conception of the work. All authors contributed to the article and approved the submitted version.

## Conflict of Interest

The authors declare that the research was conducted in the absence of any commercial or financial relationships that could be construed as a potential conflict of interest.
